# On the feasibility of a robotic probe manipulator for echocardiography in the prone position

**DOI:** 10.3389/frobt.2024.1474077

**Published:** 2024-11-18

**Authors:** Muhammad Wildan Gifari, Tomoko Machino-Ohtsuka, Takeshi Machino, Modar Hassan, Kenji Suzuki

**Affiliations:** ^1^ School of Integrative and Global Major, University of Tsukuba, Tsukuba, Japan; ^2^ Biomedical Engineering Department, Institut Teknologi Sumatera (ITERA), South Lampung, Indonesia; ^3^ Department of Cardiology, Institute of Medicine, University of Tsukuba, Tsukuba, Japan; ^4^ Institute of Systems and Information Engineering, University of Tsukuba, Tsukuba, Japan

**Keywords:** soft robots, medical robots, robotic ultrasound, prone position, echocardiography

## Abstract

Robotic probe manipulator for echocardography (echo) can potentially reduce cardiac radiologists’ physical burden. Echo procedure with industrial robots has wide Range of Motion (RoM) but poses safety risks because the robot may clamp the patient against the bed. Conversely, a soft robotic manipulator for echo has safe contact force but suffers from a limited RoM. Due to COVID-19, cardiac radiologists explored performing echo in the prone-positioned patients, which yielded good-quality images but was difficult to perform manually. From robot design perspective, prone position allows safer robot without clamping issue because all actuators are under the patient with minimal RoM to reach the cardiac windows. In this work, we propose a robotic probe manipulator for echo in the prone position employing a combination of a delta 3D printer and a soft end-effector and investigate its feasibility in a clinical setting. We implemented the robot as a scanner type device in which the probe manipulator scans from under a bed with an opening around the chest area. The doctor controls the robot with a joystick and a keypad while looking at a camera view of the chest area and the ultrasound display as feedback. For the experiments, three doctors and three medical students scanned the parasternal window of the same healthy subject with the robot and then manually. Two expert cardiologists evaluated the captured ultrasound images. All medical personnel could obtain all the required views with the robot, but the scanning time was considerably longer than the manual one. The ultrasound image quality scores of the doctors’ group remained constant between manual and robotic scans. However, the image scores of the robotic scan were lower in the students’ group. In summary, this work verified the ability to obtain clinically sufficient images in echocardiography in the prone position by expert medical doctors using the proposed robotic probe manipulator. Our robot can be further developed with semi automatic procedure to serve as a platform for safe and ergonomic echocardiography.

## 1 Introduction

An echocardiography (echo) or cardiac ultrasound (US) requires tiresome probe manipulation. First, the cardiac radiologists bend and extend their arms to find the cardiac window - a small opening between the ribs from which the probe ultrasound wave could reach the heart. Then, they adjust the view angle to locate a specific part of the heart. Adjusting the angle requires wrist rotation, probe gripping, hand flexion or extension, and US screen monitoring simultaneously. Because cardiac radiologists are limited specialists, they have to perform these demanding tasks during most of their working day, which contributes to the work-related musculoskeletal disorders (WRMSD) (Harrison and Harris, 2015). A recent survey of 152 cardiac radiologists in Saudi Aabia found that WRMSD prevalence is as high as 84.8% in which 63.2%, 55.9%, 51.3%, and 23% of the cohort reported pain on their shoulders, hands, necks, and elbows, respectively (Al Saikhan, 2023). Similar prevalence of WRMSD among cardiac radiologists were reported by a similar multi-site cross-sectional study in the USA (Barros-Gomes et al., 2019). Countermeasures to reduce the burden, such as scanning with both hands (Seto and Biclar, 2008) and stretch exercise (Christenssen, 2001) have been suggested, but their effectiveness is still uncertain.

To reduce cardiac radiologists’ risk of WRMSD, ideally an automatic echo scanner similar to a mammography device is desirable. However, recent survey of Robotic Ultrasound (RUS) by (von Haxthausen et al., 2021) pointed that currently only teleoperated, collaborative, and partly autonomous robot had been implemented. As RUS manipulators physically interact with the patient’s body, safely controlling the force while performing the probe manipulation is the most important aspect. Mathiassen et al. (2016) proposed compliance force control and haptic teleoperation with UR5 (Universal Robots, Odense, Denmark) lightweight industrial robot platform and tested them on a phantom. Fang et al. (2017) used the same UR5 platform in a co-robotic system to assist the doctor’s hand during scanning. Phantom testing showed human force reduction from 20 N to 2–13 N with the system (Fang et al., 2017). Although feasibility of UR5 for US procedures was recently verified in patient testing (Solvin et al., 2023), conducting subject testing poses a safety risk due to the possibility of the robot excessively presses the patient against the bed in a clamping scenario (Haddadin et al., 2008). demonstrated that when the robot presses the subject against a constraint, excessive pushing force even with low velocity can cause lethal injury.

To address the safety issue (Arent et al., 2016;Arent et al., 2017) developed the Remote Medical Diagnostician (ReMeDi) robot - a mobile robot with an arm-type lightweight manipulator teleoperated with a haptic device. Remote echo trials of the ReMedi second prototype involved eight doctors and 14 healthy persons, in which the doctors could obtain images from all cardiac windows except the suprasternal one because it was outside the ReMeDi arm workspace (Giuliani et al., 2020). However, the study did not compare the image quality of the remote exam with the traditional exam. Another robot, Medirob, also employed a lightweight manipulator and teleoperation with force feedback (Boman et al., 2009). The clinical feasibility of Medirob for tele-echography in rural settings was demonstrated (Boman et al., 2014). Currently, Medirob has been commercialized (RoboCraft-InnovationR, 2024). Another safety solution by MELODY, also a commercialized robot, is to have a human assistant adjust the robot translation and compression force while a remote cardiac radiologist is controlling the other DoFs (Adechotech, 2024; Gourdon et al., 1999; Nouaille et al., 2010; Krupa et al., 2016). Using this approach, the robot does not need force sensors but a human assistant beside the patient is necessary. To summarize, a lightweight robotic arm with haptic teleoperation or manual force control is a feasible solution for the robotic US.

On the other hand (Lindenroth et al., 2017; 2020), employed a compliant Soft Parallel Actuator (SPA) manipulator to obtain images from various view planes of a fetal phantom. The teleoperation of SPA offered safe interaction without force control. However, the system had only three soft actuators with a Range of Motion (RoM) of 22.3 mm in XYZ and 14.02 in tilt (Lindenroth et al., 2020), which is only enough to adjust the view planes after arriving at the target location. In addition, the SPA did not have a DoF for rotation. That means a human assistant or a passive arm must manually position and orient the SPA toward the target location before a cardiac radiologist remotely controls the SPA toward a specific view plane. Although excellent in terms of safety, the movement range of soft actuators is limited.

During an echo procedure, patients lay on their side in the Left Lateral Decubitus (LLD) position to move the heart closer to the chest wall by gravity and decrease the interposed lung volume (Mitchell et al., 2019). However, because of the need to ventilate the COVID-19 patients (Griffiths et al., 2019; Papazian et al., 2019), multiple medical groups started to explore the possibility of echocardiography from the prone position (Roemer et al., 2020; Gibson et al., 2020; Cheong et al., 2022). Roemer et al. (2020) reported the feasibility of obtaining Right Ventricle (RV), Apical 4-chambers, apical long-axis, apical 2-chambers, and transhepatic Inferior Vena Cava (IVC) views as 100%, 95,8%, 79.2%, 45.8%, and 33.3%, respectively, from a cohort of 24 subjects without respiratory diseases. The images were sufficient to calculate RV-related echo parameters such as RV longitudinal strain. However, due to interference with the operation table, (Roemer et al., 2020) could not obtain the parasternal view. Similarly, (Gibson et al., 2020) obtained RV, apical, and IVC views with sufficient quality for cardiac assessment from 27 patients with Acute Respiratory Distress Syndrome (ARDS) but could not obtain the parasternal view. Interestingly, (Gibson et al., 2020) indicated the superiority of the apical 4-chambers view obtained with the prone position in obese patients compared to the supine position. Other study by (Cheong et al., 2022) reported the possibility of getting cardiac function measurements from an apical view in the prone position among ARDS patients. Although a few clinical studies have confirmed the feasibility of manually obtaining images in the prone position (Roemer et al., 2020; Gibson et al., 2020; Cheong et al., 2022), there is not yet a RUS that utilizes the prone position.

**FIGURE 1:**
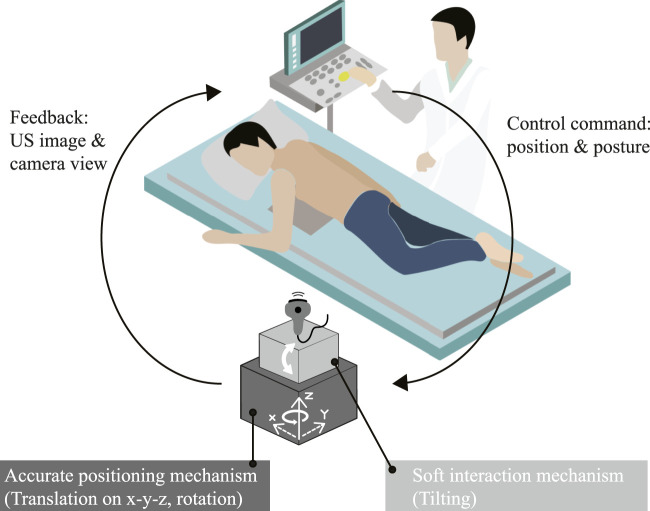
Operational concept of the proposed prone-position based robotic probe manipulator for echocardiography examination.

All the previous robots scan the patients who are lying down in the supine or the LLD posture. If the robot scans from the top (Mathiassen et al., 2016; Fang et al., 2017), then the patient is at risk of getting clamped (Haddadin et al., 2008). Scanning from the side of a patient who is lying down (Arent et al., 2016; 2017; Giuliani et al., 2020) requires the robot to have an extensive RoM for reaching a location far from the robot’s trunk. Ensuring safety by delegating some of the robot DoFs to human operator (Adechotech, 2024) hinders the robot autonomy. Based on the RUS literature covered and as mentioned by (von Haxthausen et al., 2021), the missing step toward a fully autonomous RUS is a robotic platform with a reliable movement and safety strategy in a closed-loop control. We hypothesize that utilizing prone position to design a robot that scan from below the patient could eliminate the risk of the patient getting clamped while allowing robot design with minimum RoM to reach the cardiac windows without the need of a human assistant (Figure 1). In our concept, because the robot is centered at the chest it only needs half of the lateral RoM of a robot which scans from the side. Moreover, we expect that such prone-position based robot could obtain clinically sufficient images. In our previous work, we proposed a mechanical design of prone-position based RUS employing a combination of stepper motors and a soft end-effector (Gifari et al., 2022b). However, the robot did not have an integrated control interface. In this work, we proceed to a teleoperated robot system with a user interface capable of acquiring medially located parasternal window views in a clinical setting. The contributions of this paper are.

• Hardware and control interface of a bed-type robotic scanner for echo in the prone position.

• Controlled experiments in which medical students and doctors operated the robot to acquire various views of the parasternal window from a healthy subject lying in the prone position.

• Time performance, image evaluation, and questionnaire scores of robotic versus manual scan comparing the students and doctors group.

## 2 Materials and methods

### 2.1 Robot hardware

**FIGURE 2:**
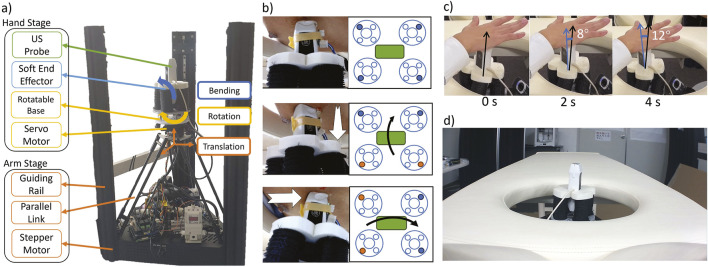
**(A)** two-stage implementation of the proposed prone-position-based echocardiography robot, **(B)** (top) schematic implementation of the probe position. Each big circle at the side is an individual actuator (see Fig 3.5 in Gifari (2018). Blue-filled circles are the chambers connected to the air control system. (middle) The schematic of the probe tilts along its long axis and (bottom) along its short axis. Orange-filled circles are the actuated chambers. **(C)** Time clip of the probe tilt around its contact point. The angle is measured with ImageJ software Schneider et al. (2012) **(D)** A bed with a chest opening from which the robot scans the patient.

Figure 2A shows the detailed implementation. The robot has a two-stage design: 1) the “arm” stage to move the probe horizontally and vertically (3 DoFs) and 2) the “hand” stage to rotate and tilt the probe (3 DoFs). In the arm stage, three stepper motors of a delta 3D printer (FLSUN Super Racer, FLSUN, China) connected to three links with guiding rails translate the robot in the *X*-*Y*-*Z* directions. In the previous work, the robot could reach a RoM of a 22 cm diameter circle Gifari et al. (2022b). In this work, we upgraded the 3D printer to (FLSUN Super Racer, Zhengzhou Chaokuo, China), so the X-Y range increased to 26 cm diameter to cover the chest area better. We flipped the 3D printer upside down so its extruder base pointed upward. Then, we removed the printing base at the top of the 3D printer to remove obstacles in the upward probe movement. In the hand stage, we replaced the printer extruder with a 3D-printed base rotated by a servo motor (LSS-HT1, LSS, USA) which was confirmed to achieve 90° rotation range with enough precision Gifari et al. (2022b). On top of the rotatable base, we installed a soft end-effector assembled from four soft actuator (SA) modules arranged in two antagonistic pairs (Figure 2B). The fabrication method of individual modules can be referred to in our previous works in which each module could exert 3 N and bend 30° at 0.3 kPa Gifari (2018); Naghibi et al. (2019). To satisfy force output requirements at the required bending degree in Gifari et al. (2022b), a pair of actuators is installed for each bending direction. Two pairs of antagonistic SA modules enable independent control of the bending movement around the *X* and *Y*-axes in both positive and negative directions.

The end-effector controller consists of a digital pressure regulator (ITV 2030, SMC, Japan) and four solenoids (030E1, Koganei, Japan) connected to each of the four SAs. The regulator actuates two adjacent SAs simultaneously to maximize the bending force (Figure 2B middle and bottom). The elongation of the SA during bending increases the contact force and prevents slip from the skin. In addition, the rotatable base held by stepper motors prevents the soft end-effector from moving laterally and vertically. As a result, the probe will be tilted at the skin contact point as its fulcrum (Figure 2C). In the previous prototype, we confirmed that the SA end-effector fulfills the contact force and RoM requirements of 13.57 ± 0.20 N and 22.8 ± 0.76° , respectively Gifari et al. (2022b). Finally, we put the robot under a bed with an opening in the chest area where the robot moves upward to scan the patient in the prone position (Figure 2D).

### 2.2 Teleoperated control scheme

Similar to the teleoperation of Lindenroth et al. (2020) SPA, we did not measure nor use any force sensing in our control implementation. However, we are ensuring robot force safety from four aspects: end-effector compliance, upward control scheme, maximum force threshold, and prone-position design. The robot end-effector consisted of soft actuators with a stiffness of 0.17 N/mm Gifari et al. (2022a), which is comparable to the human skin stiffness of 0.047–0.118 N/mm Boyer et al. (2007). In addition, the upward probe movement from the stepper motor is realized in a 1-mm step as instructed by the joystick button press, so there would not be any sudden jump in the thrust force. If a high contact force occurs, the 3D printer stepper motors will automatically disengage if its force threshold of 52.7 ± 1.7 N is achieved. This threshold is still below the safe limit for painful pinching contact of 70 N for sternum and 60 N for pectoral muscle Behrens et al. (2022). Finally, in the case when the patient starts to feel uncomfortable during the scanning, due to the prone position robot setup the patient can easily escape from the bed.

**FIGURE 3:**
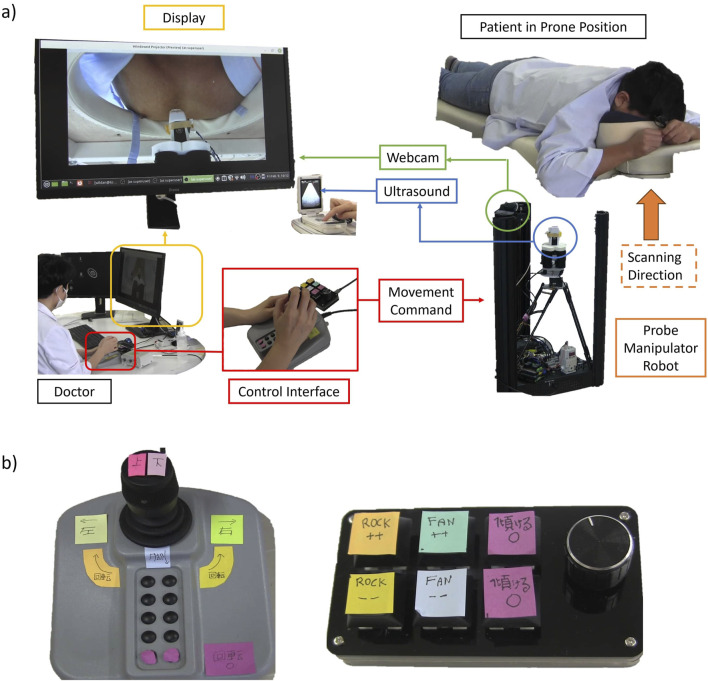
**(A)** Diagram of the Human in the Loop control. The user commands the robot with a joystick and a keypad while having a webcam stream and live US images as feedback. **(B)** Close-up look at the joystick and the keypad interface.

Figure 3A shows the Human-in-the-Loop control diagram of our robot. The operator decides the next control command based on the chest’s webcam view and the US live feed (GE Vscan, GE, Norway). The chest’s view is to infer the probe position and rotation angle relative to the chest. Because there is no force feedback, the US image acts as the operator’s surrogate to estimate the current contact force in addition to deduce which heart structure the probe is currently viewing at, similar to what the operator does in the manual scanning. The operator then moves the robot’s 6 DoF with a joystick (IP Desktop, APEM, USA) and a 6-button keypad interface (Figure 3B). Data communication between the control interface and the robot is via the ROS2 Foxy topic framework.

For the control command, the joystick is for probe XYZ movement and rotation. Moving the joystick to the front moves the robot horizontally toward the head. Moving it to the back moves the robot toward the legs. Similarly, the left and right movement of the joystick will move the robot to the left and right, respectively. Rotating the joystick rotates the robot in the same direction. We assigned a home button to return the rotation angle to the initial position. The keypad is for controlling the probe tilt. Each key press increases the bending angle incrementally. In this way, the operator does not need to hold the key, so he can focus on monitoring the US images. We assigned one pair of buttons for rocking in positive and negative directions and another pair for fanning in positive and negative directions. Rocking is tilting along the probe’s short axis, and fanning is tilting along the probe’s long axis. In the current implementation rocking and fanning cannot occur concurrently because the controller only has one pressure regulator.

### 2.3 Experiment setup

We obtained ethical consent from the Faculty of Engineering, Information, and Systems, University of Tsukuba (approval number 2022R716). Prior to the experiments, we took informed consents from all the participants, including consent to publish identifiable images or videos. We recruited six medical personnel: three medical students, and three expert cardiologists. All medical personnel scanned the same healthy male participant (age = 31, Body Mass Index = 25) to avoid bias in the scanning time and the image results. The medical personnel’s task is to obtain five distinct views from the parasternal axis, both manually and using the robot. The views are: Parasternal Long Axis (PLAX), Parasternal Short Axis (PSAX) aortic valve (AV) level, PSAX mitral valve (MV) level, PSAX papillary muscle (PM) level, and PSAX apical level. We chose the parasternal window as the testbed because it was difficult to obtain in manual prone position scanning (Roemer et al., 2020; Gibson et al., 2020). We picked the five views because the user must move the robot in all DoFs (translation, rotation, and tilting) to obtain them.

A cardiac doctor (TM, one of the authors) was present in all the experiments and guided medical student participants in obtaining the images. Each medical personnel performed a 1-h session. First, the medical personnel had a video explanation about obtaining the cardiac views manually and how to control the robot, followed by a practice session of robot scanning with a phantom. Then, they performed cardiac scanning for all five views with the robot. Before the robotic scan, the doctor (TM) marked the parasternal window position at the patient’s chest with a red marker. After the robotic scanning, they performed manual scanning for all five views. We recorded short clips (cine-loop) from the US probe at the starting time and the arrival of each view and measured the time to reach the first view and the total time of each scanning procedure.

### 2.4 Evaluation criteria

To compare the scanning performance and assess the effort of finding the parasternal window, we calculated the ratio of the time to get the first clinically meaningful image (time to the window) to the total scanning time for each participant’s manual and robotic scanning. We call this ratio the operational cost. For example, in the robotic scanning, P1 time to window was 12 min while P1 total scanning time was 37 min. Therefore, the operational cost for P1 in the robotic scanning was 12/37 = 33%.In addition, we checked how many of the five required parasternal views each participant could obtain.

We asked two expert cardiologists to rate the quality of the cine-loop images. The image metrics were: Clearness, Adequate for diagnosis, Cross-sectional shape, and Position. Clearness and cross-sectional shape criterion are similar to the clarity and foreshortedness criterion of Labs et al. (2023). We used a five-point Likert scale for each item. We described the criterion at the beginning of the image evaluation form. “Clearness” is defined as how clear are the cardiac structures, such as ventricular walls and valves. “Adequate for diagnosis” is explained as: Can you diagnose an abnormality or determine that the heart is healthy from this echo image? The “Cross-sectional Shape” is how close the image is to the standard cross-sectional echocardiogram. For example, if the left ventricular short-axis image is not a regular circle but an oval due to an oblique slice, it is an inappropriate cross-section. “Position” is defined as how close the echo image is to the center of the field of view. The two experts rated 60 cine-loops, which consist of five views from two types of scanning (robotic and manual) times six participants. The evaluation took place in a Google form in which the experts did not know whether the images were from robotic or manual scanning.

After the scanning, we inquire about how many years the medical personnel have performed echocardiography. Then, two questionnaires are given. One is the Nasa Task Load Index (TLX), a subjective evaluation to assess the task load Hart and Staveland (1988). The medical personnel filled each of the TLX questions with a rating from 1 (very low) to 10 (very high) for both the manual and robotic scan. The other questionnaire is an inquiry about the comfort and ease of use of the robot. As in the previous questionnaire, the medical personnel should answer with a rating from 1 (strongly disagree) to 10 (strongly agree) to these questions.

1. I feel comfortable in using the robot
2. My hand feels less tired compared to the manual scanning
3. The robot’s ability to hold its position reduces my workload
4. I feel confident that the robot will not hurt my patient
5. The robot is easy to operate
6. I can easily aim a target cardiac view using the robot
7. The robot’s fine movement is small enough so I can precisely target a cardiac view
8. I do not feel mentally exhausted using the robot during the experiment
9. I feel confident that the robot follows my command

## 3 Results

### 3.1 Scanning performance

**FIGURE 4:**
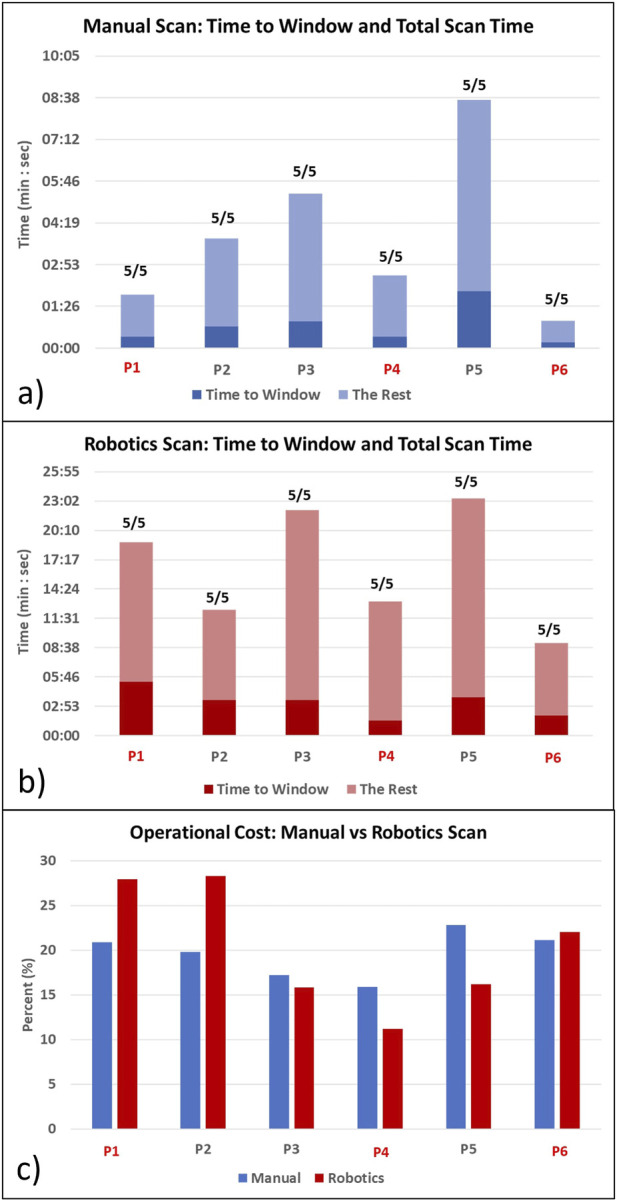
Time to get the first clinically meaningful image (Time to Window) and time to manipulate the probe after arriving at the window (the Rest) for **(A)** manual scanning and **(B)** robotic scanning. The ratio on the top of the bar indicates how many obtained views out of the five target views. Total scanning time is Time to Window + The Rest. **(C)** Operational cost comparison of manual vs. robotic scanning. Red colors denote the doctor participants.

**FIGURE 5:**
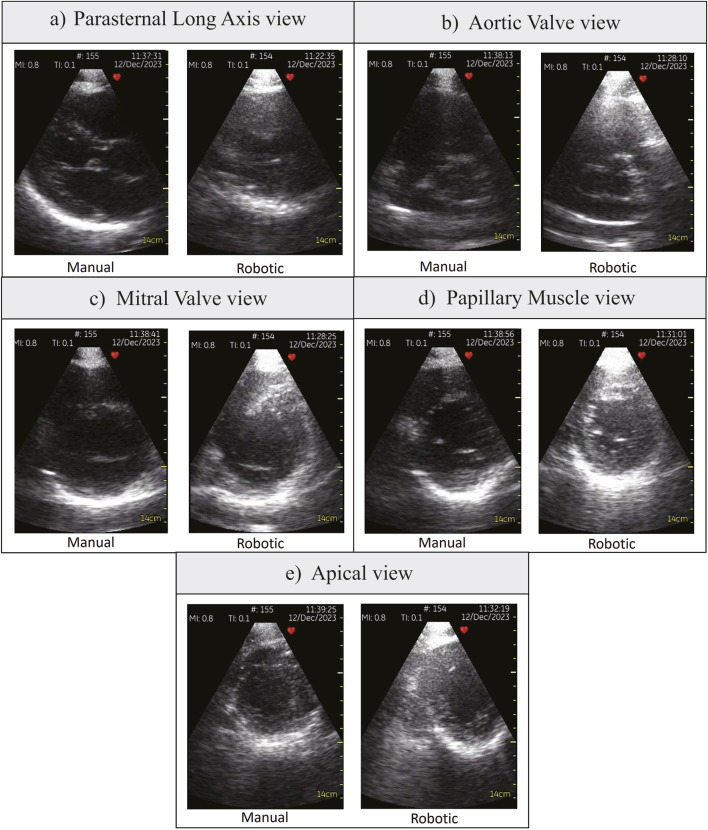
Various parasternal views obtained by participant P4, a doctor. **(A)** Parasternal Long Axis view, **(B)** Aortic Valve view, **(C)** Mitral Valve view, **(D)** Papillary Muscle view, **(E)** Apical view.

Table 1 shows the statistical analysis comparing all participants’ robotic and manual scanning performance and dividing them into students and doctors groups. Figures 4A, B show the time to the window and the total scanning time of the manual and robotic exams, respectively. The number on top of the bar chart is a ratio of the obtained views out of the five required views. All the required views were obtained by all the operators both with the robot and manually. Figure 5 presents examples of the five views obtained with the robot and manually from participant P4, a doctor.

**TABLE 1:** Statistical analysis of scanning performance among all participants and comparing students vs. doctors group. Time to Window and Total Time are the average in min: sec. Operational Costs are the average in %. Sig. = significant (*) or not (n). The statistical test for overall manual versus robotic is a 2-tailed paired *t*-test, α = 0.1. The test for students versus doctors groups is a 2-tailed unpaired *t*-test, α = 0.1.

Parameter	Overall			Manual			Robotic		
	Manual	Robotic	Sig	Students	Doctors	Sig	Students	Doctors	Sig
Time to Window	00:46	03:16	*	01:12	00:20	*	03:35	02:56	n
Total Time	03:50	16:31	*	05:54	01:46	*	19:16	13:46	n
Operational Cost	19.60	20.23	n	19.92	19.29	n	20.11	20.36	n

Comparing students and doctors groups in the manual scanning (Figure 4A; Table 1 Manual column), doctors’ time to the window and total time was nearly four times faster than students. However, in the robotic scanning (Figure 4B; Table 1 robotic column), there was no significant difference in time to window and total time between the two groups. Averaging all participants, the time to the window and total time increased by nearly four times in the robotic scanning (Table 1 Overall column). Because both times increased in the same proportion, the operational cost which is the ratio between the two stayed the same at around 20% (Table 1 Overall column). In addition, Figure 4C shows that there is no clear trend in the operational cost. Some participants’ operational costs were lower with the robotic device, but other participants’ operational costs were higher.

### 3.2 Image evaluation

**FIGURE 6:**
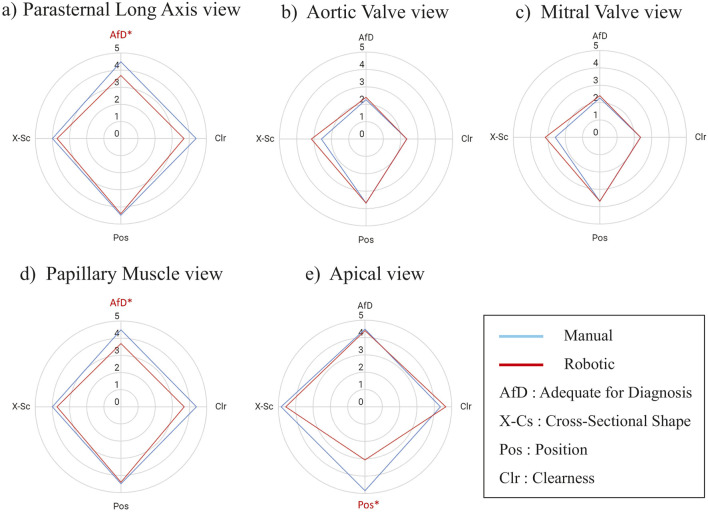
Score Comparison (mean) of robotic vs. manually scanned images for each of the parasternal window views in all participants. The images are evaluated according to these metrics: AfD = Adequate for Diagnosis, X-Cs = Cross-Sectional Shape, Pos = Position, Clr = Clearness. The value is a five points Likert scale. An asterisk and red color mark a metric with a significant difference. The statistical test was a two-tailed Wilcoxon signed rank with α = 0.05. **(A)** Parasternal Long Axis view, **(B)** Aortic Valve view, **(C)** Mitral Valve view, **(D)** Papillary Muscle view, **(E)** Apical view.

Figure 6 compares the image evaluation scores for each of the parasternal views. In the AV, MV, and PM views, the image scores of robotic scanning across the views were not different from manual scans (Figures 6B–D). Significant differences occurred in Adequate for Diagnosis (AfD) criteria in the PLAX view (Figure 6A), with the robotic scanning score 0.83 points lower. The Clearness score is also 0.75 points lower in the robotic scan, although not significant (Figure 6A). In the Apex view (Figure 6E), the Position score was considerably 1.75 points lower in the robotic scanning.

**FIGURE 7:**
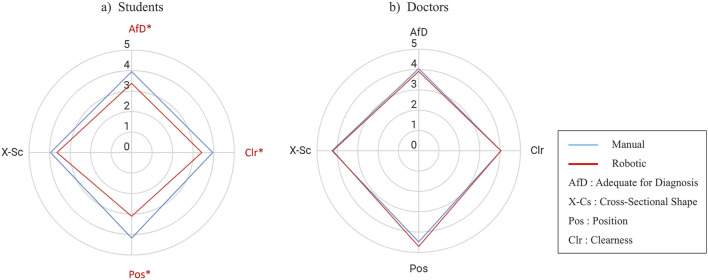
Score comparison (mean) of the four evaluation metrics for robotic vs. manually scanned images in **(A)** students group and **(B)** doctors group. The value is a five points Likert scale. An asterisk and red color mark a metric with a significant difference. The statistical test was a two-tailed Wilcoxon signed-rank with α = 0.05.

Figures 7A, B present scores of image evaluation in the students’ and doctors’ groups, respectively. In the students’ group, the image scores significantly differed in Position, Clearness, and AfD criterion, in which the Position had the highest mean difference of 1.07 points (Figure 7A). On the other hand, doctors’ image qualities seemed robust without any significant difference in all the evaluation criterion (Figure 7B).

### 3.3 Questionnaire answers

**FIGURE 8:**
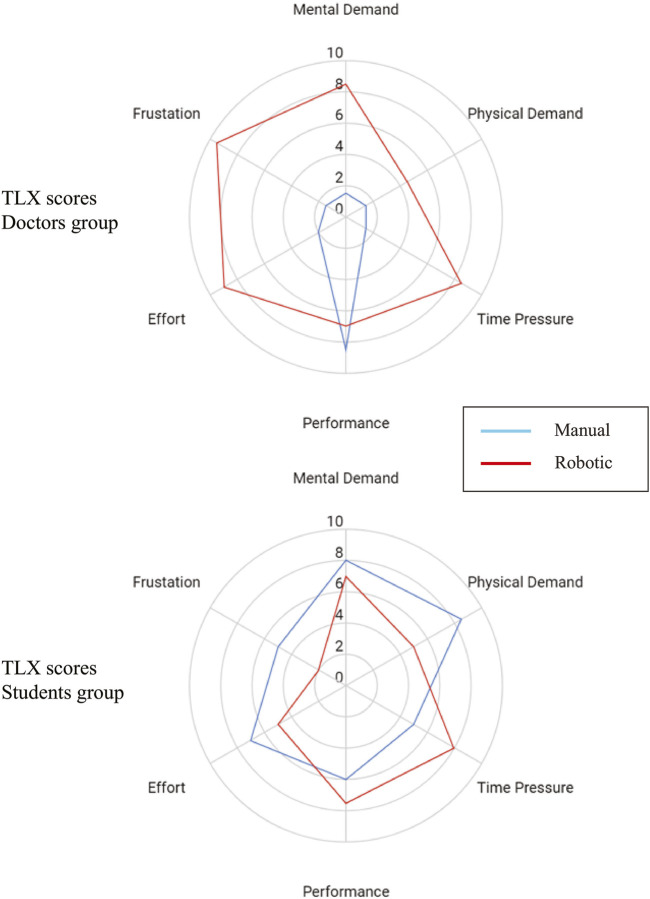
TLX Score comparison (median) of manual vs. robotic scanning procedure in (top) doctors group and (bottom) students group.

Figure 8 shows the TLX score from the doctors group and the students group comparing the manual and robotics scan. Two of the doctors and two of the students filled the TLX questionnaire. Statistical tests were not performed due to the small amount of samples. However, from the score we can infer several points. First, the doctors perceived that their performance did not decrease much with the robot, while the other aspects increased. Second, the students perceived that using the robot they could perform better than manually, while having less effort, frustration, mental, and physical demand. Third, the students and doctors both perceive the same level of physical demand, time pressure, and performance with the robot, with the value of 5,8, and 7, respectively.

**FIGURE 9:**
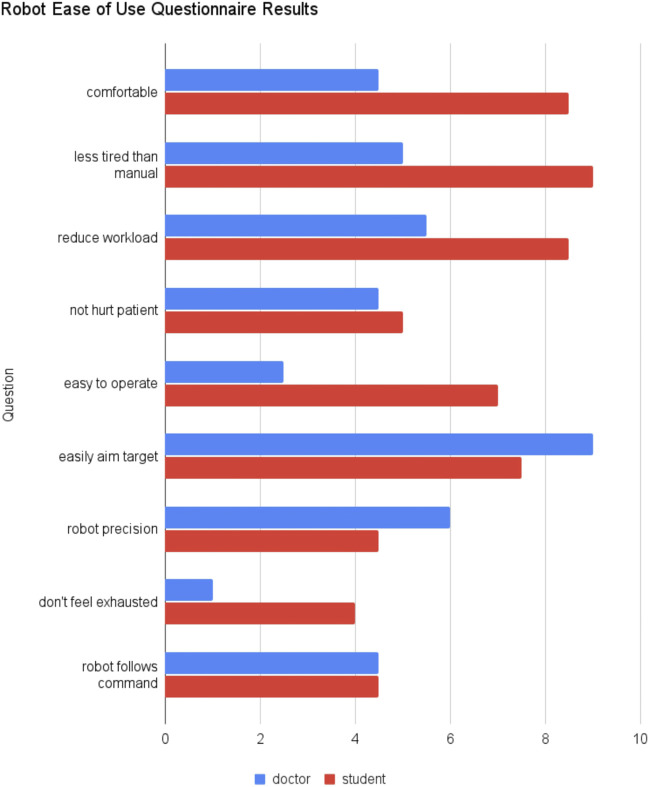
Robot ease of use questionnaire results comparing the median of doctors and students group.

Figure 9 shows the robot ease of questionnaire score from the doctors group and the students group. As in the previous questionnaire, two of the doctors and two of the students returned the form. Due to the limited sample, we did not perform any statistical test. The students feel that the robot is more comfortable, less tired than manual scan, reduces workload, and easier to operate than the doctors feel. In addition, the students did not feel as exhausted in using the robot as the doctors did. Both the students and doctors are not so sure whether the robot will not hurt patient and if the robot follows the command. Interestingly, the doctors agree that the robot can easily aim at targets with considerable precision more than the students.

## 4 Discussion

Our primary aim with the prone position design is to build a robot with reliable movement and safety experience in a closed-loop control. First, we will discuss the robot safety strategy in the image acquisition context. Generally, the patient felt comfortable during the scanning as the patient was able to fall asleep during a few of the trials. Sometimes, uncomfortable scanning forces did happen, so the patient just said it out loud and the medical personnel would lower the robot position to decrease its upward thrust. This is possible due to joystick button controlled upward movement, so the movement bandwidth is limited to the frequency of button stroke. There was never any case in which the robot disengaged due to the stepper motors threshold achieved. All medical personnel could obtain all the required views using the robot, with the doctors’ image scores stayed constant compared to the manual scan (Figure 7). Solvin et al. (2023) also performed teleoperated echo trials using industrial robot UR5 with the control algorithm described in Mathiassen et al. (2016). Their safety strategy was to limit the contact force to 5 N and retract the robot if this limit is reached. However, during their experiments, the force limit could be incidentally reached due to the jittery probe movement Solvin et al. (2023). If it happened, the robot would withdraw and the operator would need to repeat the image acquisition movement sequences, which makes fine probe adjustment more tedious. As a result, the robotic exam image scores were 1.6 on average in contrast to the manual scan score of 2.5 in 0–3 Likert scale (Figure 4 in Solvin et al. (2023)). In rigid robots, such concurrent adaptive force and orientation control could be attained using hierarchical approach Santos and Cortesão (2018), which resulted in accurate orientation tracking. However, the setup needed force sensing, depth camera, and online stiffness estimation which has significant computational cost. Compared to a rigid robot, a compliant end-effector has small force gradation due to its low stiffness, so it does not need a precise force tracking algorithm with high bandwidth. Because the patient can tolerate force increment during tilting, we could implement a step-wise increment of actuator bending without force sensing. Using this approach, the doctors could obtain images from various view planes with similar quality to the manual scan (Figure 5).

Another benefit of a soft end-effector with bending motion supported by stepper motors base is the ability to execute a tilting movement while pressing simultaneously about a fulcrum located in the skin contact point (Figure 2C). The supporting base function of the motors is possible because of the prone position. If the robot scans from the side or the top, it will be difficult to establish a stable base for the soft end-effector. Solvin et al. (2023) mentioned the difficulty to isolate tilting movements after arriving at a window as all UR5 joints must move simultaneously. Custom motorized robot for remote shoulder US using circular rail guides proposed by Koizumi et al. (2009) have the advantage of isolating the tilting movement around a fulcrum, but in echo only tilting without applying pressure could make the probe loses its contact pressure, which causes the probe to lose the US image. This problem of losing contact in circular rail design was also present in the body mounted teleoperated probe manipulator in Krupa et al. (2016) because of its light mass. To maintain contact in the body mounted probe holder, Ito et al. (2013) utilized pre-tensioned mechanical spring. Despite its effectiveness to stabilize the contact, the force applied was not adjustable during the scanning because the spring is always in tension. On the other hand, by changing the air pressure of a soft actuator, we can adjust the contact force and bending angle simultaneously Gifari et al. (2022b). Still, the amount of allowable bending becomes smaller when the contact force is higher Lindenroth et al. (2020) in a nonlinear manner which renders it difficult to use a kinematic based approach for such a soft actuator. However, in our teleoperated scheme the doctor knows if the required bending and force is achieved through the US image so US based feedback could potentially bypass solving inverse kinematics of the robot.

Previously, Gibson et al. (2020) noted the positive effect of prone position to acquire clearer image in several cases of obese patients. In addition, CT and MRI measurement shows that prone position brings the heart closer to the chest wall with less interposed lung volume compared to supine position Chino and Marks (2008). From the physics perspective, prone position should be better than the Left Lateral Decubitus (LLD) to bring the heart closer to the thorax because the gravity vector is more orthogonal to the chest wall in the prone position. The question we had is ‘why prone echo scanning is not common?’ We argued that this is due to the difficulty to do prone position scanning with manual setup. However, a robotic manipulator such as the proposed robot can easily perform prone scanning to reach the medially located parasternal window which is difficult to reach in manual prone scanning Roemer et al. (2020). Although the results from our image evaluation in the healthy patient could not confirm the superiority of prone position scanning, we expect that in the future prone-based robots could facilitate clinical studies to compare prone versus traditional position effect to echocardiography images. At least, at this point, based on the literature Roemer et al. (2020); Gibson et al. (2020); Cheong et al. (2022) and our results, there is no significant negative effect of prone position to the image quality.

Despite the fact that the doctors’ image quality was preserved, the robotic scanning time for the doctors group increased by 9-fold (Table 1). In the experiments, the healthy subject did not report any discomfort due to the scanning time, since he was laying comfortably in the prone position. Still, there are other scenarios in which remote scanning even with longer time than manual one could be useful, such as for rural patients who need to travel to a primary hospital Boman et al. (2014) or for an emergency patient to receive remote echo inside an ambulance on the way to a hospital. In the future, we expect that improvement in the robotic aspect and incorporation of AI assistance would reduce the robotic scanning time.

Because the robotic scanning was a new experience for the students and the doctors, the difference in the manual scanning time between the students’ and the doctors’ groups did not carry over in the robotic scanning (Table 1). With the robot, the students could perform at a similar time performance with the doctors. Dividing time to first image and total scanning time, we got the operational cost value, which indicates the effort to find the first window. If the participants had difficulty finding the first window, the operational cost will be high. Due to an indication from Chino and Marks (2008) that it could be easier to find the window in the prone position, we expected that the operational cost should be lower. However, our results (Table 1) pointed to a similar value of operational cost between manual and robotic scanning, which indicated that medical personnel could find the window with the same relative effort as the manual scan. We could argue that while it may be easier to find the window in the prone position, the unfamiliarity with the robotic control interface caused more difficulty in finding the window. In the future, it is interesting to check the operational cost for manual prone scanning to better verify this argument.

Considering Figures 6, 7, the students had lower score in the robotic scan in the PLAX and the Apex views. Specifically, they had difficulty obtaining clear PLAX images, which resulted in a low AfD score. As the echo experiment was the first time for the students and the PLAX view was their first image, they were still learning how to adjust the probe pressure to get a clear echo image. The low Clearness and AfD score may arise from the difficulty in adjusting the probe contact to obtain the PLAX view. The students also had low score in the position aspect in the Apex view. In the positioning of the probe, one doctor (TM) was present with the students during the scanning and guided them on where to move the probe. For the manual scanning, TM could guide the students’ hand on where and how to move. However, for the robotic scanning, TM had difficulty translating the required probe movement into the robot movement. As a result, the students only knew where to move but had to infer how to move the probe. This difficulty is more pronounced in the Apex view because acquiring this view needs positioning and tilting at the same time. For example, if the image is centered while the cross section is oblique, then when the tilt is applied, the cross section will be circular but the image is not centered in the frame. In the experiments, the students (with TM guidance) tend to optimize the cross section with the cost of missing centering the image in the US frame. On the other hand, the doctor participants knew what kind of probe position and orientation they wanted to achieve, and they could perform trial and error to translate the target probe pose into the required probe movements. These results emphasize the importance of AI guidance to help non-experts automatically adjust the probe position and orientation to assist in obtaining clearer images.

The TLX questionnaire showed intriguing results. While the Doctors’ TLX with manual scan is typical for experts (very sharp point in performance aspect), in the robotic scan their perceived performance did not decrease sharply, which is *confirmed* by image analysis results. On the other hand, students perceived that their performance increased with the robotic scan, in addition to lower scores in effort, frustration, mental demand, and physical demand. However, image analysis results showed that students’ image performance *actually decreased*. The students’ bias that they performed better could be because they are more relaxed in the other aspects of the robotic experiments (effort, frustration, mental demand, and physical demand). Interestingly, both students and doctors perceived high time pressure with the robotic device. This could mean that the spacing between each robot commands was dense as the robot control became intense especially when it almost arrived at the target view. In the future, automation to optimize the cross section Jiang et al. (2022) or the target view position Soemantoro et al. (2023) may help reduce the time pressure. The robot ease of use questionnaire showed a similar trend. The students perceived the robot as more comfortable, less tired, and easy to operate. But, interestingly, the doctors perceived that they can easily aim the target with good precision with the robot. On the other hand, the doctors feel very exhausted in using the robot. Improving the control interface and incorporating some automation in the workflow may improve these aspects.

## 5 Conclusion

We developed a robotic probe manipulator for echocardiography in the prone position and tested it in a clinical setting with different medical personnel and one healthy subject. All medical personnel could acquire all five required views from the medially located parasternal window with the robot. Although the robotic scanning took longer, the operational cost did not differ, which implies that it is not more difficult to find the window than in the manual scan. In addition, the image evaluation scores showed that the image quality from the robotic scan did not differ from the manual scan in the doctors’ group. However, in the students’ group, the image quality was significantly lower. The experts user need better interface and automation to reduce their frustation, effort, mental and physical demand. As the students perceived the system to be comfortable and less tiring, it may be suitable to use the platform as echo training system.

In conclusion, these results confirmed that expert medical doctors could perform an echo procedure from the prone position with the teleoperation of the proposed robot to obtain clinically sufficient images, even in their first attempts to use the robot with less than 10 min of training. To the best of our knowledge, this is the first evidence of using a robotic probe manipulator for echocardiography in the prone position in subject testing. We indicated that the robot has a reliable safety strategy to acquire images from various view planes in a human-in-the-loop control. By incorporating AI assistance, we expect to pave a way for an automatic echo scanning device similar to a mammography scanner.

## Data Availability

The raw data supporting the conclusions of this article will be made available by the authors, without undue reservation.

## References

[B1] Adechotech (2024). Melody, a remote, robotic ultrasound solution. Available at: https://www.adechotech.com/products/ (Accessed July 15, 2024).

[B2] Al Saikhan L. (2023). Prevalence, characteristics, consequences, and awareness of work-related musculoskeletal pain among cardiac sonographers compared with other healthcare workers in Saudi Arabia: a cross sectional study. PLOS ONE 18 (5), 1–19. 10.1371/journal.pone.0285369 PMC10162565 37146012

[B3] Arent K. Cholewiński M. Chojnacki Ł. Domski W. Drwiega M. Jakubiak J. (2017). Selected topics in design and application of a robot for remote medical examination with use of ultrasonography and auscultation from the perspective of the remedi project. J. Automation, Mob. Robotics Intelligent Syst. 11, 82–94. 10.14313/JAMRIS_2-2017/20

[B4] Arent K. Jakubiak J. Drwiega M. Cholewiński M. Stollnberger G. Giuliani M. (2016). “Control of mobile robot for remote medical examination: design concepts and users’ feedback from experimental studies,” in 2016 9th international conference on human system interactions (HSI), 76–82.

[B5] Barros-Gomes S. Orme N. Nhola L. Scott C. Helfinstine K. Pislaru S. (2019). Characteristics and consequences of work-related musculoskeletal pain among cardiac sonographers compared with peer employees: a multisite cross-sectional study. J. Am. Soc. Echocardiogr. 32, 1138–1146. 10.1016/j.echo.2019.04.416 31227328

[B6] Behrens R. Pliske G. Umbreit M. Piatek S. Walcher F. Elkmann N. (2022). A statistical model to determine biomechanical limits for physically safe interactions with collaborative robots. Front. Robotics AI 8, 667818. 10.3389/frobt.2021.667818 PMC8850785 35187090

[B7] Boman K. Olofsson M. Berggren P. Sengupta P. P. Narula J. (2014). Robot-assisted remote echocardiographic examination and teleconsultation: a randomized comparison of time to diagnosis with standard of care referral approach. JACC Cardiovasc. Imaging 7, 799–803. 10.1016/j.jcmg.2014.05.006 25124011

[B8] Boman K. Olofsson M. Forsberg J. Boström S.-r. (2009). Remote-controlled robotic arm for real-time echocardiography: the diagnostic future for patients in rural areas? Telemedicine e-Health 15, 142–147. 10.1089/tmj.2008.0079 19292622

[B9] Boyer G. Zahouani H. Le Bot A. Laquieze L. (2007). “ *In vivo* characterization of viscoelastic properties of human skin using dynamic micro-indentation,” in 2007 29th annual international conference of the (IEEE Engineering in Medicine and Biology Society), 4584–4587. 10.1109/IEMBS.2007.4353360 18003026

[B10] Cheong I. Otero Castro V. Gómez R. A. Merlo P. M. Tamagnone F. M. (2022). Transthoracic echocardiography of patients in prone position ventilation during the covid-19 pandemic: an observational and retrospective study. Int. J. Cardiovasc. Imaging 38, 2303–2309. 10.1007/s10554-022-02659-z 36434340 PMC9244514

[B11] Chino J. P. Marks L. B. (2008). Prone positioning causes the heart to be displaced anteriorly within the thorax: implications for breast cancer treatment. Int. J. Radiat. Oncology*Biology*Physics 70, 916–920. 10.1016/j.ijrobp.2007.11.001 18262103

[B12] Christenssen W. D. (2001). Stretch exercises: reducing the musculoskeletal pain and discomfort in the arms and upper body of echocardiographers. J. Diagnostic Med. Sonogr. 17, 123–140. 10.1177/87564790122250318

[B13] Fang T.-Y. Zhang H. K. Finocchi R. Taylor R. H. Boctor E. M. (2017). Force-assisted ultrasound imaging system through dual force sensing and admittance robot control. Int. J. Comput. assisted radiology Surg. 12, 983–991. 10.1007/s11548-017-1566-9 28343302

[B14] Gibson L. E. Di Fenza R. Berra L. Bittner E. A. Chang M. G. (2020). Transthoracic echocardiography in prone patients with acute respiratory distress syndrome: a feasibility study. Crit. Care Explor. 2, e0179. 10.1097/cce.0000000000000179 32832914 PMC7417147

[B15] Gifari M. W. (2018). Study on the design of soft surgical robots for endoscopic NOTES application. The Netherland: University of Twente. Master’s thesis. Available at: http://essay.utwente.nl/76537/

[B16] Gifari M. W. Hassan M. Suzuki K. (2022b). “Teleoperated probe manipulator for prone-position echocardiography examination,” in 2022 44th annual international conference of the IEEE engineering in medicine and biology society (EMBC), 4350–4353. 10.1109/EMBC48229.2022.9871021 36086338

[B17] Gifari M. W. Hassan M. Suzuki K. (2022a). “Design of probe manipulation robot for echocardiography,” in *The Proceedings of JSME annual Conference on Robotics and mechatronics (robomec)* 2022, 1P1–M07. 10.1299/jsmermd.2022.1P1-M07

[B18] Giuliani M. Szcześniak-Stańczyk D. Mirnig N. Stollnberger G. Szyszko M. Stańczyk B. (2020). User-centred design and evaluation of a tele-operated echocardiography robot. Health Technol. 10, 649–665. 10.1007/s12553-019-00399-0

[B19] Gourdon A. Poignet P. Poisson G. Vieyres P. Marche P. (1999). “A new robotic mechanism for medical application,” in 1999 IEEE/ASME international conference on advanced intelligent mechatronics (cat. No.99TH8399), 33–38.

[B20] Griffiths M. J. D. McAuley D. F. Perkins G. D. Barrett N. Blackwood B. Boyle A. (2019). Guidelines on the management of acute respiratory distress syndrome. BMJ Open Respir. Res. 6, e000420. 10.1136/bmjresp-2019-000420 PMC6561387 31258917

[B21] Haddadin S. Albu-Schaffer A. Frommberger M. Hirzinger G. (2008). The role of the robot mass and velocity in physical human-robot interaction - part ii: constrained blunt impacts. IEEE Int. Conf. Robotics Automation, 1339–1345. 10.1109/robot.2008.4543389

[B22] Harrison G. Harris A. (2015). Work-related musculoskeletal disorders in ultrasound: can you reduce risk? *Ultrasound* 23 10.1177/1742271X15593575 PMC4760593 27433262

[B23] Hart S. G. Staveland L. E. (1988). “Development of nasa-tlx (task load index): results of empirical and theoretical research,” in North-holland, vol. 52 of *Advances in psychology* . Editors Mental Workload H. Hancock P. A. Meshkati N., 139–183. 10.1016/S0166-4115(08)62386-9

[B24] Ito K. Sugano S. Takeuchi R. Nakamura K. Iwata H. (2013). Usability and performance of a wearable tele-echography robot for focused assessment of trauma using sonography. Med. Eng. and Phys. 35, 165–171. 10.1016/j.medengphy.2012.04.011 22613671

[B25] Jiang Z. Li Z. Grimm M. Zhou M. Esposito M. Wein W. (2022). Autonomous robotic screening of tubular structures based only on real-time ultrasound imaging feedback. IEEE Trans. Industrial Electron. 69, 7064–7075. 10.1109/TIE.2021.3095787

[B26] Koizumi N. Warisawa S. Nagoshi M. Hashizume H. Mitsuishi M. (2009). Construction methodology for a remote ultrasound diagnostic system. IEEE Trans. Robotics 25, 522–538. 10.1109/TRO.2009.2019785

[B27] Krupa A. Folio D. Novales C. Vieyres P. Li T. (2016). Robotized tele-echography: an assisting visibility tool to support expert diagnostic. IEEE Syst. J. 10, 974–983. 10.1109/JSYST.2014.2314773

[B28] Labs R. B. Vrettos A. Loo J. Zolgharni M. (2023). Automated assessment of transthoracic echocardiogram image quality using deep neural networks. Intell. Med. 3, 191–199. 10.1016/j.imed.2022.08.001

[B29] Lindenroth L. Housden R. J. Wang S. Back J. Rhode K. Liu H. (2020). Design and integration of a parallel, soft robotic end-effector for extracorporeal ultrasound. IEEE Trans. Biomed. Eng. 67, 2215–2229. 10.1109/tbme.2019.2957609 31804926 PMC7115900

[B30] Lindenroth L. Soor A. Hutchinson J. Shafi A. Back J. Rhode K. (2017). “Design of a soft, parallel end-effector applied to robot-guided ultrasound interventions,” in 2017 IEEE/RSJ international conference on intelligent robots and systems (IROS), 3716–3721.

[B31] Mathiassen K. Fjellin J. E. Glette K. Hol P. K. Elle O. J. (2016). An ultrasound robotic system using the commercial robot ur5. Front. Robotics AI 3. 10.3389/frobt.2016.00001

[B32] Mitchell C. Rahko P. Blauwet L. e. a. Canaday B. Finstuen J. A. Foster M. C. (2019). Guidelines for performing a comprehensive transthoracic echocardiographic examination in adults: recommendations from the american society of echocardiography. J. Am. Soc. Echocardiogr. 32, 1–64. 10.1016/j.echo.2018.06.004 30282592

[B33] Naghibi H. Gifari M. W. Hoitzing W. Lageveen J. W. van As D. M. M. Stramigioli S. (2019). “Development of a multi-level stiffness soft robotic module with force haptic feedback for endoscopic applications,” in International conference on robotics and automation, ICRA 2019 (Montreal, QC, Canada), 1527–1533. *May 20-24, 2019* (IEEE).

[B34] Nouaille L. Smith-Guérin N. Poisson G. Arbeille P. (2010). Optimization of a 4 dof tele-echography robot. IEEE/RSJ International Conference on Intelligent Robots and Systems, 3501–3506.

[B35] Papazian L. Aubron C. Brochard L. Chiche J. D. Combes A. Dreyfuss D. (2019). Formal guidelines: management of acute respiratory distress syndrome. Ann. Intensive Care 9, 69–2309. 10.1186/s13613-019-0540-9 31197492 PMC6565761

[B36] RoboCraft-Innovation, R (2024). Medirob tele echocardiography robot. Available at: https://www.medirob.com/en/tele.php (Accessed December 01, 2023).

[B37] Roemer S. Kaminski A. Payne A. Tanel E. Perez Moreno A. C. Jaglan A. (2020). Feasibility of transthoracic imaging of the heart in the prone position. J. Am. Soc. Echocardiogr. 33, 1147–1148. 10.1016/j.echo.2020.07.004 32891256 PMC7373037

[B38] Santos L. Cortesão R. (2018). Computed-torque control for robotic-assisted tele-echography based on perceived stiffness estimation. IEEE Trans. Automation Sci. Eng. 15, 1337–1354. 10.1109/TASE.2018.2790900

[B39] Schneider C. A. Rasband W. S. Eliceiri K. W. (2012). Nih image to imagej: 25 years of image analysis. Nat. Methods 9, 671–675. 10.1038/nmeth.2089 22930834 PMC5554542

[B40] Seto E. Biclar L. (2008). Ambidextrous sonographic scanning to reduce sonographer repetitive strain injury. J. Diagnostic Med. Sonogr. 24, 127–135. 10.1177/8756479308315230

[B41] Soemantoro R. Kardos A. Tang G. Zhao Y. (2023). An ai-powered navigation framework to achieve an automated acquisition of cardiac ultrasound images. Sci. Rep. 13, 15008. 10.1038/s41598-023-42263-2 37696901 PMC10495422

[B42] Solvin H. Sajadi S. Lippert M. Massey R. Holmstrøm H. Elle O. (2023). Feasibility of teleoperated robotic echocardiography – a pilot study. WFUMB Ultrasound Open 1, 100018. 10.1016/j.wfumbo.2023.100018

[B43] von Haxthausen F. Böttger S. Wulff D. Hagenah J. García-Vázquez V. Ipsen S. (2021). Medical robotics for ultrasound imaging: current systems and future trends. Curr. Robot. Rep. 2, 55–71. 10.1007/s43154-020-00037-y 34977593 PMC7898497

